# Enteral Nutrition in Dementia: A Systematic Review

**DOI:** 10.3390/nu7042456

**Published:** 2015-04-03

**Authors:** Joanne Brooke, Omorogieva Ojo

**Affiliations:** 1 Kent Community Health NHS Trust, The Oast, Unit D, Maidstone Kent ME16 9NT, UK; 2 School of Health and Social Care, Faculty of Education and Health, University of Greenwich, Avery Hill Campus, London SE9 2UG, UK; E-Mail: o.ojo@greenwich.ac.uk

**Keywords:** enteral nutrition, dementia, percutaneous endoscopic gastrostomy, nasogastric tube, serum albumin

## Abstract

The aim of this systematic review is to evaluate the role of enteral nutrition in dementia. The prevalence of dementia is predicted to rise worldwide partly due to an aging population. People with dementia may experience both cognitive and physical complications that impact on their nutritional intake. Malnutrition and weight loss in dementia correlates with cognitive decline and the progress of the disease. An intervention for long term eating difficulties is the provision of enteral nutrition through a Percutaneous Endoscopic Gastrostomy tube to improve both nutritional parameters and quality of life. Enteral nutrition in dementia has traditionally been discouraged, although further understanding of physical, nutritional and quality of life outcomes are required. The following electronic databases were searched: EBSCO Host, MEDLINE, PubMed, Cochrane Database of Systematic Reviews and Google Scholar for publications from 1st January 2008 and up to and including 1st January 2014. Inclusion criteria included the following outcomes: mortality, aspiration pneumonia, pressure sores, nutritional parameters and quality of life. Each study included separate analysis for patients with a diagnosis of dementia and/or neurological disease. Retrospective and prospective observational studies were included. No differences in mortality were found for patients with dementia, without dementia or other neurological disorders. Risk factors for poor survival included decreased or decreasing serum albumin levels, increasing age or over 80 years and male gender. Evidence regarding pneumonia was limited, although did not impact on mortality. No studies explored pressure sores or quality of life.

## 1. Introduction

Due to a global aging population the incidence and prevalence of dementia is predicted to rise worldwide, with an estimated 81 million people diagnosed with dementia by 2040 [1]. Dementia is an umbrella term for a number of specific conditions which are progressive in nature and impact on multiple areas of functioning including decline in memory, reasoning, communication skills and ability to carry out daily activities [2].

A common experience for people with dementia is the development of eating difficulties leading to problems such as malnutrition and weigh loss [3,4]. The severity and progression of dementia is closely related to weight loss [5,6]. In the early stages of dementia eating difficulties are attributed to olfactory and taste dysfunction, executive planning difficulties, attention deficits, dyspraxia, agnosia and behavioural problems [7]. In advanced stages of dementia oral and pharyngeal phase dysphagia may be present leading to the inability to coordinate chewing and swallowing, and disruption of the food bolus from the oropharynx into the oesophagus without aspiration [8]. Reduced nutrition has negative outcomes for patients with dementia including higher morbidity and mortality, reduced quality of life and increased carer burden [9,10,11].

An intervention for long term eating difficulties across different health conditions is the provision of enteral nutrition through a Percutaneous Endoscopic Gastrostomy (PEG) tube. The provision of enteral nutrition is both to provide complete nutrition and improve the patient’s quality of life [12]. A systematic review in 1999 explored the impact of enteral nutrition in patients with advanced dementia and found no improvements in the rates of aspiration, pressures sores or mortality [13]. No data was found on quality of life, although many complications were reported including: gastric perforation, gastric prolapse, aspiration, diarrhoea, gastrointestinal bleeding, nauseas and vomiting, fluid overload and loss of social aspects of feeding. Finucane *et al.* [13] concluded enteral nutrition for patients with dementia should be actively discouraged.

A further review in 2001 explored nutritional parameters, quality of life and mortality of older people with dementia receiving enteral nutrition [14]. A small number of studies (*n* = 3) found improvements in nutritional parameters and an increased albumin was associated with decreased mortality. Dharmarajan *et al.* [14] found quality of life was difficult to analyse in this population as patients with advanced dementia could not narrate their subjective feelings, and family members reported conflicting opinions. Mortality ranged from 11%–27% across studies at 30 days post insertion of a PEG and commencement of enteral nutrition. However, mortality was not uniformed: older patients, men and patients with an acute illness had higher mortality than women and African-American patients. Dharmarajan *et al.* [14] recommended caution in decisions regarding enteral nutrition in older people with dementia.

A more recent review in 2009 reported no significant association between enteral nutrition and decreased mortality in older patients with dementia [15]. Secondary outcomes of weight loss, Body Mass Index (BMI), haemocrit and cholesterol were not significantly different between those receiving enteral nutrition to those who were not, albumin levels were significantly decreased in patients receiving enteral nutrition [15]. No studies in this review explored the impact of enteral nutrition on quality of life, behavioural or psychiatric symptoms of dementia. However, one study documented the use of restraint, with 71% of patients being physically restrained to prevent removal of a PEG compared to 55% of those not receiving enteral nutrition [16]. Sampson *et al.* [15] conclude that there is insufficient evidence to suggest enteral nutrition in patients with advanced dementia is beneficial.

Clinical guidance reflects the evidence to date, National Institute for Health and Care Excellence (NICE) guidance [17] suggests enteral nutrition may be considered if dysphagia in a patient with dementia is deemed to be transient, but should not generally be used for patients with advanced dementia who are disinclined to eat or have permanent dysphagia. European Society of Parenteral and Enteral Nutrition (ESPEN) are shortly to realise their guidelines on Nutrition in Dementia [7]. ESPEN confirm the use of enteral nutrition in patients with mild or moderate dementia if malnutrition is predominantly the cause of a reversible condition and only for a limited time. Reversible conditions are secondary concurrent illnesses such as depression, infection, over use of sedatives, pain or poor oral health. ESPEN do not recommend the use of enteral nutrition in the terminal phase of dementia, although acknowledge decisions are unique for each patient with dementia and should take into consideration the patient’s general prognosis and preferences.

Decisions regarding enteral nutrition in advanced dementia remain ethically challenging for all involved [4]. One challenge is the possible complications of enteral nutrition including aspiration pneumonia and fluid overload [13,18]. Further challenges include understanding the patient’s wishes as they may be unable to communicate and are unlikely to have documented their wishes through advance directives or advance care plans [19,20].

However, evidence suggests the continued use of enteral nutrition in the older population with dementia. A study in the United States found 34% of nursing home residents received enteral nutrition [21] and 30% of PEG insertions were estimated to be in people with dementia [22]. In Japan, elderly people receiving enteral nutrition is on the increase [23]. Many studies and reviews have been completed exploring the immediate clinical effects of enteral nutrition for people with advanced dementia [13,14,15,24]. However, methodologies, focus and outcomes of these studies have begun to change and the need to explore further risk factors for patients with dementia receiving enteral nutrition is required. Therefore the aim of this review is to explore recent data on both physical and nutritional outcomes and the impact on quality of life of patients with dementia receiving enteral nutrition.

Objectives:

- Evaluate the impact of enteral nutrition on mortality, risk factors for mortality, pressure sores, aspiration pneumonia and nutritional parameters for patients with dementia.
- Evaluate the impact of enteral nutrition on quality of life for patients with dementia.

## 2. Experimental Section

Published guidelines [25,26] were used to complete a systematic review. An initial scoping exercise identified three relevant systematic reviews [13,14,15], which informed the criteria for the search. The following electronic databases were searched: EBSCO Host, MEDLINE, PubMed, Cochrane Database of Systematic Reviews and Google Scholar for publications from 1st January 2008 and up to and including 1st January 2014. Search words included *enteral nutrition*, *enteral feeding*, *artificial nutrition*, *artificial nutrition*, *percutaneous endoscopic gastrostomy* and *dementia*, with *and/or* Boolean operators (refer to Table 1 Literature Search Strategy). All searchers were limited to “English Language”. In addition bibliographies of identified articles were manually searched for relevant studies.

**Table 1:** Literature Search Strategy.

Key Words	Search Engine	Hits	Search Engine	Hits	Search Engine	Hits	Search Engine	Hits	Search Engine	Hits
enteral nutrition ‘and’ dementia	EBSCO Host	168	PubMed	100	MEDLINE	317	COCHRANE DATABASE	3	GOOGLE SCHOLAR	5630
enteral feeding ‘and’ dementia	EBSCO Host	62	PubMed	102	MEDLINE	324	COCHRANE DATABASE	3	GOOGLE SCHOLAR	4380
enteral feeding ‘and’ dementia patients	EBSCO Host	13	PubMed	63	MEDLINE	324	COCHRANE DATABASE	3	GOOGLE SCHOLAR	4510
artificial nutrition ‘and’ dementia	EBSCO Host	96	PubMed	39	MEDLINE	98	COCHRANE DATABASE	1	GOOGLE SCHOLAR	14,300
nasogastric tube ‘and’ dementia	EBSCO Host	14	PubMed	0	MEDLINE	38	COCHRANE DATABASE	2	GOOGLE SCHOLAR	16,100
percutaneous endoscopic gastrostomy ‘and’ dementia	EBSCO Host	2	PubMed	78	MEDLINE	124	COCHRANE DATABASE	2	GOOGLE SCHOLAR	2330
artificial feeding ‘and’ dementia	EBSCO Host	30	PubMed	354	MEDLINE	947	COCHRANE DATABASE	1	GOOGLE SCHOLAR	18,500

### 2.1. Inclusion and Exclusion Criteria

In addition to the search strategy above the inclusion criteria were: measured outcomes of mortality, aspiration pneumonia, pressure sores, nutritional parameters and quality of life, and a separate analysis of patients with a primary diagnosis of dementia or neurological disease. Exclusion criteria were: administration of enteral nutrition via nasogastric tubes, intravenous fluids and short term interventions. Two studies were excluded as analysis combined the outcomes of patients with dementia receiving enteral nutrition via a nasogastric tubes and PEG tubes [27,28].

## 3. Results

A total of five studies were included in the systematic review and all had an observational design. Two studies applied a prospective design [29,30] and three studies applied a retrospective design [31,32,33]. Studies were completed in Japan [31], USA [32], Sweden [29,33] and Germany [30]. Study sample sizes ranged from 119–484, participants were categorized with a diagnosis of dementia [31,33] or a broader definition of dementia as significant cognitive impairment and/or combined with other neurologic disorders [29,30,32]. All studies included enteral nutrition administered via PEG tubes. All studies included mortality following the commencement of enteral nutrition or the insertion of a PEG. Mortality was analysed using Kapan-Meirer analysis, which is an estimate of the number of participants who survive for a certain amount of time following a healthcare intervention [34]. Secondary outcomes included predictors of mortality, serum albumin levels, aspiration pneumonia and general complications. No studies reported outcomes relevant to pressure sores or quality of life (refer to Table 2 Summary of studies reviewed).

### 3.1. Mortality

Kaplan-Meirer survival analysis were completed by all five studies [29,30,31,32,33]. No significant differences in mortality were demonstrated in two studies when patients with dementia were compared to those without dementia or other neurological conditions [31,32,33]. Decreased mortality for patients with dementia was demonstrated by one study when compared to patients with stroke, malignant diseases and other neurological conditions [33]. Increased mortality for patients with dementia and other neurological diseases was a significant finding in two studies [20,30] when compared to patients with tumours.

### 3.2. Predictors of Mortality

A low or decreasing serum albumin was a predictive factor of increased mortality in three studies [29,31,32]. Increasing age, or age over 80 years were predictive factors of increased mortality in four studies [29,31,32,33]. Further risk factors identified by individual studies included male [31], an additional diagnosis of chronic heart failure [31] and a raised CRP [29].

### 3.3. Pressure Sores

No studies included in the review explored the impact of enteral nutrition and pressure sore development and healing.

### 3.4. Aspiration Pneumonia

Pneumonia including aspiration pneumonia was explored by three studies [30,31,32]. Rates of pneumonia whilst receiving enteral nutrition via a PEG tube was 5% and was not a risk factor of mortality [30,31,32]. One study reported aspiration pneumonia rates were comparable across patients with dementia and those without dementia receiving enteral nutrition [31]. One study reported pneumonia rates, not linked to aspiration were comparable across patients with neurologic conditions and tumours receiving enteral nutrition. 

### 3.5. Quality of Life

No studies included in the review explored the impact of enteral nutrition and quality of life for patients with dementia. 

**Table 2:** Summary of Studies Reviewed.

Author	Study, Design, Country of Study	Population Size	Age Mean SD	Kaplan-Meier Survival Analysis	Predictors for Poor Survival
Higaki *et al.* 2008 [31]	Retrospective study of PEG enteral nutrition Compared outcomes of patients with and without dementia in the elderly Japan	311 46.0% (*n* = 143) with dementia 54.0% (*n* = 168) without dementia 78.8	83.7 ± 8 with dementia 78.8 ± 11 without dementia	No significant difference in mortality between patients with dementia and those without dementia (*p* = 0.62)	-subtotal gastrectomy (OR 2.619, 95% CI: 1.367–5.019) -serum albumin < 2.8 g/dL (OR 2.081, 95% CI: 1.490–2.905) -age > 80 years (OR 1.721, 95% CI: 1.234–2.399) -chronic heart failure (OR 1.541, 95% CI: 1.096–2.168) -male (OR 1.407, 95% CI: 1.037–1.909)
Gaines *et al.* 2009 [32]	Retrospective study of PEG enteral nutrition Compared outcomes for patients with dementia or significant cognitive impairment (SCI) to those without these conditions USA	190 23.7% (*n* = 45) dementia or SCI 76.3% (*n* = 145) without dementia or SCI	Median age: 64	No significant difference in mortality in patients with dementia or SCI and those without (*p* = 0.85)	Predictors for 30-day mortality -increasing age (OR 1.08, 95% CI: 1.04–1.12) -decreasing serum albumin (OR 0.43, 95% CI: 0.22–0.84)
Malmgren *et al.* 2011 [33]	Retrospective study of PEG enteral nutrition Indications for survival after PEG insertion in patients older than 65 Sweden	191 8.4% (*n* = 16) dementia 5.8% (*n* = 11) Parkinson 9.5% (*n* = 19) miscellaneous 49.7% (*n* = 95) stroke 18.4% (*n* = 35) malignant 6.8% (*n* = 13) neurological diseases	79.0 ± 7	Patients with dementia or Parkinsons had longest median survival	-patients with dementia >80 years of age than those with dementia <80 years of age (*p* = 0.025)
Blomberg *et al.* 2012 [29]	Observational prospective study of PEG enteral nutrition Outcome of patients following PEG insertion Sweden	484 44% (*n* = 214) tumours 45% (*n* = 218) neurological disease including dementia	66.0 ± 14	Mortality higher in patients with neurological disorders than those with tumours (*p* = 0.002)	-serum albumin < 30 g/L (hazard ration (HR), 3.46; 95% CI 1.75–6.88) -CRP ≥ 10 (HR, 3.47; 95% CI 1.68–7.18) -age ≥ 65 (HR, 2.26; 95% CI 1.20–4.25)
Schneider *et al.* 2014 [30]	Observational prospective study of PEG enteral nutrition Outcomes of patients following PEG insertion Germany	119 57.2% (*n* = 68) tumours 29.4% (*n* = 35) neurologic including dementia 13.4% (16) other	63.0 ± 13	Mortality higher in patients with neurological disorders than those with tumours (*p* = 0.002)	NA

## 4. Discussion

In this review, the impact of enteral nutrition on mortality was equivalent for patients with dementia, without dementia or diagnosed with other neurological conditions. However, patients with dementia had decreased mortality compared to patients with a stroke and increased mortality compared to patients with tumours. Risk factors for poor survival included decreased or decreasing serum albumin levels, increasing age or over 80 years, and male gender. Limited evidence on pneumonia was found, although did not impacted on mortality. No studies explored the development or healing of pressure sores or quality of life.

Previous studies have failed to demonstrate enteral nutrition for patients with dementia prolongs survival [18,35,36,37]. Current studies suggest mortality of patients with dementia receiving enteral nutrition when compared to other conditions is dependent on the comparativeness of these conditions, including stage of the disease and long term prognosis. The importance of the timing of the decision with regards to the prognosis of the patient with dementia may be an influential factor, as enteral nutrition is more frequently commenced in advanced dementia [33]. Patients with advanced dementia may exhibit a low level of functionality over a long period of time, which contributes to general frailty [38]. Illness trajectories and mortality in dementia are difficult to predict due to low functionality and frailty, which leads to discussions regarding enteral nutrition in the advanced stages of dementia as end of life is difficult to recognize [38,39]. Therefore, the possibility of some studies to include older patients with more advanced dementia and the tendency to commence enteral nutrition in the late stages of the disease process may have implications for mortality rates.

In the current review a decreased or decreasing serum albumin was a predictor of mortality [29,31,32]. Decreased serum albumin levels (<3.0 mg/dL) have been associated with increased mortality in enteral nutrition where analysis did not differentiate the diagnosis of patients [40,41]. Evidence for the impact of the diagnosis of dementia and decreasing serum albumin levels for patients receiving enteral nutrition is inconsistent. One study found serum albumin levels did not predict survival in patients with dementia, but did predict survival in patients without dementia receiving enteral nutrition [42]. The impact of serum albumin levels in patients not receiving enteral nutrition needs to be considered, as decreased serum albumin in critical illness was associated with increased mortality [43]. In the healthy elderly serum albumin levels decreased with age and were predictive of mortality independent of know disease [44]. Evidence regarding decreased or decreasing serum albumin levels suggests an impact on mortality and therefore, needs to be considered in the provision of enteral nutrition regardless of diagnosis but with consideration of age.

Aspiration pneumonia has been a recognised complication of advanced dementia and enteral nutrition administered via PEG tubes [3]. Finucane *et al.* [13] reported no randomised controlled trials had explored the reduction of aspiration pneumonia following the provision of enteral nutrition via a PEG tube. In the current review one observational study reported aspiration pneumonia occurrence at 5%, which was comparable for patients with and without dementia and was not a risk factor of mortality [31]. Tentatively enteral nutrition delivered through a PEG tube does not increase the risk of aspiration for patients with dementia compared to rates of aspiration pneumonia of other disease cohorts.

The development and healing of pressure sores was not explored by the studies included in this review. However, Martin *et al.* [27] explored the impact of enteral nutrition administered via nasogastric tubes and PEG tubes reported pressure and reported that a fifth of patients developed a pressure sore during the provision of enteral nutrition, and the healing of pressure sores was correlated with increased mortality. The development and lack of healing of pressure sores may correlate with hypoalbuminemia, as this is a risk factor for the development of pressure sores and increases resistance to treatment [45,46]. No further studies have explored the correlation between serum albumin and pressure sores in patients with dementia receiving enteral nutrition. Martin *et al.* [27] reported enteral nutrition in patients with dementia was effective in preserving but not significantly improving serum albumin levels.

Limitations of the studies included in this review need to be acknowledged. Different clinical practices and guidelines across continents may impact on the results of studies included. Practices across continents were difficult to identify only three studies reported PEG placement procedures and none clarified/defined enteral nutrition. Prevalence of enteral nutrition in patients with dementia in Japan may be higher than Western populations due to current guidelines. In Japan guidelines compiled under the supervision of the Japan Gastroenterology Endoscopy Society recommend PEG insertion for patients who cannot maintain their nutrition due to cerebrovascular disease or dementia [47]. The impact of these guidelines may be the earlier insertion of PEG tubes and the commencement of enteral nutrition in patients with dementia leading to longer survival rates [31]. Higaki *et al.* [31] reported survival at 12 months of 51% for patients with dementia of which 20% were still alive three years, compared to 41% of patients with dementia at 12 months in a study completed in Sweden [33]. However, no longitudinal data was reported outside Japan and the challenges to this guidance and Japanese health-care system reforms may impact on this prevalence [28].

Further limitations of the studies include small sample sizes and different categorization of conditions. Dementia was categorized as a separate neurological condition in some studies, but included with other neurological conditions in further studies. Diagnosis was generalised in some studies to those with and without dementia, and more detailed in further studies with all conditions categorized and therefore, direct comparison and interpretation of results is difficult.

Ethical considerations of insertion of a PEG in a patient with dementia are important. ESPEN and NICE guidelines do not recommend enteral nutrition in patients with advanced dementia and only occasionally in patients in the earlier stages of dementia. Enteral nutrition is recommended only to ensure adequate provision of nutritional needs, when under-nutrition is caused by reversible conditions other than the dementia [48]. However, diagnostic overshadowing, a tendency to attribute all symptoms to dementia thereby leaving a co-existing conditions undiagnosed has been recognised and needs to be continually challenged [49].

Alzheimer’s Society supports the importance of quality of life rather than length of life. For the person with dementia decreased quality of life has been associated with behavioural and psychological disturbances, but no associations with dysphagia or cognition has been demonstrated to date [50]. A recent a recent review by the Royal College of Physicians suggests the need for reluctance to commence enteral nutrition in dementia, however state this cannot be translated into a blanket ban [51]. A decision-making algorithm integrating medical and ethic dimensions regarding enteral nutrition in dementia has been developed and may be helpful to healthcare professionals faced with this ethical dilemma [52].

## 5. Conclusions

The studies included in this systematic review challenge the traditional view that enteral nutrition administered through a PEG tube increases mortality in patients with dementia. The recommendations from this review include the need for a holistic assessment of patients with dementia when contemplating PEG insertion and enteral nutrition. A holistic assessment would include: the patients’ diagnosis including comorbidities, current stage and impact of dementia on the need for enteral nutrition, age and nutritional parameters. The impact of enteral nutrition on quality of life for patients with dementia remains unclear, although complications are acknowledged. Enteral nutrition within end of life care is not recommended, although this review acknowledges recognising end of life within dementia is problematic. A further recommendation is early discussions with patients with dementia and their family regarding nutrition needs in advanced dementia and the documentation of the results of these discussions. However, decision making regarding PEG insertion and enteral nutrition in patients with dementia currently remains ethically challenging and should involve discussions around appropriate end of life care.
